# The Laughing Gas Trap: Subacute Spinal Degeneration with Normal B12 levels and Co-Morbid Functional Gait Disorder

**DOI:** 10.1192/j.eurpsy.2025.832

**Published:** 2025-08-26

**Authors:** A. G. Buciuc, M. Hadjikyriakou

**Affiliations:** 1 Psychiatry, University of Miami/Jackson Health System; 2 Psychiatry and Behavioral Sciences, Bruce W. Carter Department of Veterans Affairs Medical Center, Miami, United States

## Abstract

**Introduction:**

Subacute combined degeneration (SCD) of the spinal cord is a well-known neurological disorder commonly associated with vitamin B12 deficiency. However, the condition can also present in individuals with normal B12 levels, making diagnosis more difficult.

**Objectives:**

This paper aims to examine the diagnostic challenges posed by subacute combined degeneration of the spinal cord in patients with normal B12 levels. Specifically, it aims to highlight the importance of early screening for nitrous oxide use and the complexities introduced by co-occurring functional gait disorders.

**Methods:**

Literature research was conducted using PubMed databases. The following keywords were used “whippets” or “nitrous oxide” or “inhalant use disorder”, and “subacute degeneration of spinal cord” and/ or “normal B12 levels”. Furthermore, a comprehensive clinical evaluation was conducted, including a detailed inquiry into the patient’s substance use history.

**Results:**

The 56-year-old patient developed symptoms of subacute combined degeneration (SCD) despite normal B12 levels. He experienced worsening tingling sensations, starting in the extremities and moving upward, along with new gait instability requiring a cane. Initially denying substance use, he later admitted to daily nitrous oxide inhalation, which disrupts B12 metabolism and causes spinal demyelination, leading to neurological deficits even with normal B12 levels. The presence of a functional gait disorder complicated the diagnosis, but persistent questioning about substance use and recognizing the effects of nitrous oxide were key to accurate diagnosis.

**Conclusions:**

This case highlights the importance of early and comprehensive substance use screening, particularly in patients presenting with unexplained neurological symptoms. The disruption of B12 metabolism by nitrous oxide can cause significant spinal cord degeneration, even when serum B12 levels are normal. This underscores the need for detailed, persistent questioning about substance use in clinical settings, particularly for patients with risk factors for inhalant abuse. Additionally, an awareness of the multifaceted nature of functional gait disorders is essential for accurate diagnosis and optimal patient outcomes. Routine screening for nitrous oxide use and a thorough examination of gait abnormalities can aid in the timely detection and treatment of subacute spinal degeneration, preventing further neurological damage. By incorporating recommended screening questions and being vigilant about the neurological effects of nitrous oxide, clinicians can better address the diagnostic challenges of SCD and functional gait disorders.

**Disclosure of Interest:**

None Declared

